# Brain wide engraftment of human monocytes induces neurodegeneration and cognitive dysfunction

**DOI:** 10.21203/rs.3.rs-7762816/v1

**Published:** 2025-10-10

**Authors:** Mathew Blurton-Jones, Hayk Davtyan, Jean Paul Chadarevian, Anita Lakatos, Sherry Lin, Jonathan Hasselmann, Jasmine Nguyen, Duc Duong, Joia Capocchi, Alina Lahian, Kimiya Mansour, Ghazaleh Eskandari-Sedighi, Christina Tu, Sepideh Kiani Shabestari, Fang Wu, Anantharaman Shantaraman, Michael Mgerian, Tau Lim, Alan Mai, Lauren Le, Ani Agababian, Elizabeth Head, Sandeep Robert Datta, Nicholas Seyfried

**Affiliations:** University of California, Irvine; University of California Irvine; University of California Irvine; University of California, Irvine; Harvard Medical School; University of California Irvine; University of California, Irvine; Emory University; University of California Irvine; University of California Irvine; University of California, Irvine; University of California, Irvine; University of California Irvine; University of California, Irvine; Emory University; Emory University; University of California, Irvine; University of California, Irvine; University of California, Irvine; University of California, Irvine; University of California, Irvine; University of California; Harvard Medical School; Emory University School of Medicine

**Keywords:** ontogeny, microglia, monocyte, myeloid, macrophage, iPSC, pluripotent, xenotransplantation, hematopoietic stem and progenitor cells (HSPC), bone marrow transplantation (BMT)

## Abstract

Transplantation of peripherally derived myeloid cells in combination with microglia depletion is increasingly being examined as a potential therapy for various neurological disorders.^1^–^9^ However, primitive yolk sac derived microglia and definitive bone marrow derived monocytes and macrophages exhibit distinct developmental ontogenies.^10^–^12^ Important differences between murine and human myeloid cells further obscure the translational potential of this approach.^8^,^13^–^16^ To understand the impact of peripherally derived human myeloid cells on brain function and health we utilized a xenotolerant mouse model that lacks endogenous microglia. Using a combination of spatial sequencing, proteomics, histological, biochemical, and machine learning behavioral approaches we then examined the impact of brain wide engraftment of human induced pluripotent stem cell-derived microglia, which mimic the primitive ontogeny of microglia, in comparison to definitive bone marrow derived monocytes. Here we show that engraftment of human monocytes, but not microglia, promotes a chronic proinflammatory state associated with astrogliosis, demyelination, and synaptic and neuronal loss. These robust changes in myeloid cell activation state and brain homeostasis are in turn accompanied by significant impairments in cognitive function. Together with the accompanying study by Wang and colleagues, we demonstrate the critical impact of myeloid cell ontogeny on neuronal health and function, providing important implications for the rapidly progressing development and translation of brain-wide microglial replacement therapies.^1^–^5^

Recent studies have examined the therapeutic potential of bone marrow transplantation in mouse models of Alzheimer’s disease, frontotemporal dementia, and lysosomal storage diseases.^1–5^ By combining myeloablative preconditioning with microglial-depleting CSF1R antagonists and hematopoietic stem and progenitor cell (HSPC) transplantation, these studies have shown that brain wide replacement of microglia with murine bone marrow derived ‘microglia-like cells (MLCs) leads to promising reductions in disease-associated pathologies. However, prior studies have also clearly demonstrated that despite long-term brain engraftment, peripherally derived murine MLCs remain transcriptionally, morphologically, and functionally distinct from microglia.^6–9^ Given these results along with the well-established differences in microglial versus peripheral myeloid cell ontogeny,^10–12^ it is important to understand the long-term consequences of replacing microglia with MLCs. As human and mouse microglia also exhibit significant differences, especially in regard to neurodegeneration-associated genes,^8,13–16^ it is equally important to investigate the effects of brain wide human MLC engraftment in appropriate model systems before this approach is translated into clinical practice.

Here we utilized xenotolerant FIRE mice, a mouse model that enables complete humanization of the central nervous system (CNS) microglial compartment, to examine the long-term consequences of replacing endogenous microglia with definitive HSPC-derived human monocytes or induced pluripotent stem cell (iPSC)-derived microglia (iMG) that are generated via a yolk sac-like primitive hematopoietic progenitor cell (HPC) intermediate (Fig. 1a).^17^ Fourteen weeks later, brains were examined via transcriptomic, proteomic, biochemical, and histological approaches to determine the impact of myeloid cell ontogeny on brain health and function.

Together with the accompanying study by Wang et. al., we demonstrate robust and reproducible ontogeny-dependent differences in the activation state of definitive HSPC-derived monocytes versus primitive HPC derived microglia. Importantly, these ontogeny-dependent differences mimic those observed in a human neurodegenerative disease and are further accompanied by impairments in neuronal and glial homeostasis, culminating in pronounced cognitive dysfunction. Collectively, our findings suggest that transplantation of iPSC-microglia that closely mimic the primitive developmental ontogeny of human microglia could offer a safer alternative to definitive HSPC transplantation for the treatment of neurological diseases.

## Human monocyte engraftment induces a proinflammatory state

**Figure 1.**
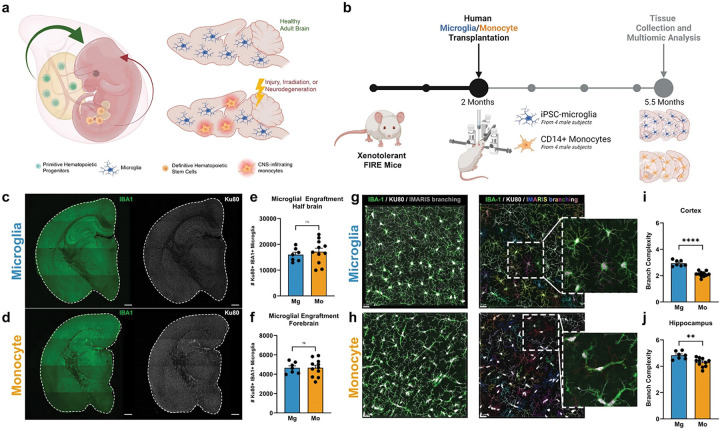
Human iPSC-microglia and blood-derived monocytes fully engraft within the brains of adult hFIRE mice but adopt differing morphologies. **(a)** Microglia arise from primitive hematopoietic stem cells within the yolk sac, whereas monocytes arise later in development from bone marrow derived definitive hematopoietic stem and progenitor cells. Lineage tracing studies have shown that very few monocytes infiltrate the healthy brain, but increased numbers migrate into the brain in response to injury, irradiation-induced loss of microglia, or neurodegeneration. **(b)** Schematic depicting transplantation paradigm of 2-month-old xenotolerant FIRE mice with iPSC-microglia (Mg) or human CD14+ monocytes (Mo) derived from 4 male subjects then sacrificed at 5.5-months of age. **(c-d)** Brain wide human (Ku80, human-specific nuclei, white) xenografted iMG (xMG) and monocytes (IBA1, green), scale bar: 500μm. **(e-f)** Quantification of xenografted microglia and monocytes in half brain coronal sections of xenotolerant hFIRE mice; *p* values from unpaired, two-sided t test. Half brain (Bregma AP −1.94): t(16)= 0.6068; ns=0.5525. Forebrain (Bregma AP +0.14): t(16)= 0.01849; ns=0.9855. **(g-h)** Representative confocal microscopy combined with IMARIS-based branching analysis of xenografted microglia and monocytes, scale bar: 20μm. **(i-j)** Quantification of xenografted microglia and monocytes branch complexity within the cortex and hippocamps; *p* values from unpaired, two-sided t test (Cortex: t(16)= 8.087; ****P<0.0001) (Hippocampus: t(16)=3.446; **P=0.0033). Data represented as average mean value ± SEM (Mg, n = 7; Mo, n = 11). ns, not significant; **p < 0.01, and ****p < 0.0001.

We previously developed xenotolerant FIRE mice that enable long-term brain wide engraftment of human microglia derived from pluripotent stem cells or surgical resection and demonstrated that engrafted iPSC-derived microglia are highly similar to brain derived human microglia.^18,19^ At 2 months of age, male mice were transplanted with human blood monocytes or iMG derived from multiple male control subjects. Fourteen weeks later, brains were examined using a combination of transcriptomic, proteomic, biochemical, and histological approaches (Fig. 1b). Both microglia and monocytes migrated well beyond the hippocampal injection site, leading to similar levels of brain wide engraftment (Fig. 1c–f). Consistent with our prior studies,^15,19^ engrafted human iMG adopted a highly ramified cell morphology consistent with the adoption of a homeostatic state (Fig. 1g). While monocytes also exhibited morphological changes with engraftment, branching complexity remained significantly lower than that of microglia and was frequently accompanied by a more elongated morphology (Fig. 1h–j).

**Figure 2.**
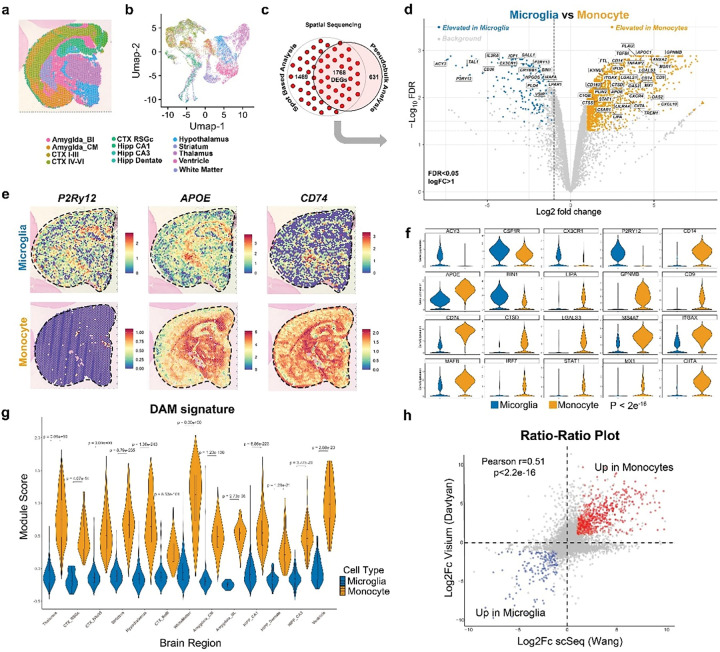
Spatial sequencing of monocyte and microglia engrafted mice reveal numerous differences in myeloid cell activation state. **(a)** Representative graph-based cluster identification from spot level (17,799 spots) computed from 10 principal components using Louvain clustering algorithm and color-coded brain segmentation. **(b)** Uniform manifold approximation and projection (UMAP) plot of analyzed spots from the brains of FIRE mice xenografted with human microglia or monocytes. **(c)** 1768 Differentially Expressed Genes (DEGs) are identified from the overlap between Spot-based and pseudobulk analysis. **(d)** Volcano plot comparing overlapping DEGs from xenografted brain sections, reveals numerous significant alterations between microglia and monocyte engrafted brains (Log_2_(FC) ≥ 1; FDR ≤ 0.05). **(e)** *P2Ry12*, *APOE*, and *CD74* mRNA expression (normalized and scaled expression) between engrafted human microglia and monocytes. **(f)** Expression level of *ACY3, CSF1R, CX3CR1, P2RY12, CD14, APOE, BIN1, LIPA, GPNMB, CD9, CD74, CTSD, LGALS3, MS4A7, ITGAX, MAFB, IRF7, STAT1, MX1, and CIITA* in brain-wide xenografted human microglia and monocytes. **(g)** Module scores of the disease-associated microglia (DAM) signature (444 upregulated genes) across segmented brain regions. *P* values in f and g were calculated by two-sided Wilcoxon test and adjusted using the Benjamini–Hochberg method. **(h)** Ratio–Ratio plot comparing the Log2Fc differences between microglia and monocytes from spatial sequencing data in comparison to scSeq data from the accompanying study by Wang et. al. Concordance between datasets was quantified via Pearson correlation coefficient (r = 0.51, p < 2 × 10^−16^).

To examine the impact of the brain environment on microglial and monocyte gene expression we utilized Visium spatial sequencing. Clustering across all six samples (n=3/group) revealed 13 regionally distinct signatures that corresponded to well established anatomical regions including upper and lower cortical layers, hippocampal subfields, the amygdala, hypothalamus, and white matter tracts (Fig. 2a,b). To maximize rigor, we focused our analysis on the intersection of differentially expressed genes (DEGs) detected using both a spot-based and pseudobulk analyses of the dataset (Fig. 2c). The use of xenotolerant FIRE mice that lack microglia coupled with a human targeted Visium probe set provided additional confidence that the great majority of DEGs detected in this experiment were derived from engrafted human myeloid cells. Consistent with this expectation, pseudobulk comparison between microglia and monocyte-engrafted tissue slices detected 1768 DEGs (LogFc>1, FDR<0.05), many of which represented well-established myeloid genes (Fig. 2d, Supplementary Table 1). While microglial engrafted tissue exhibited high levels of canonical homeostatic microglial genes (*P2RY12, SALL1, CX3CR1, VSIR*), monocyte-engrafted mice instead exhibited greatly increased expression of genes associated with proinflammatory activation of macrophages such as G*PNMB, CD9, APOE, LGALS3, STAT1, CD163,* and *CTSD* (Fig. 2d). Spatial maps of *P2RY12, APOE,* and *CD74* demonstrate the broadly increased expression of *P2RY12* in microglial samples in contrast to the greatly increased expression of *APOE* and *CD74* in monocyte engrafted brains (Fig. 2e). Spatial mapping of representative genes across all six samples further demonstrated the consistent enrichment of homeostatic microglial genes (*P2RY12, SALL1*) versus monocyte/macrophage associated genes (*CD14, APOE, CD74, MX1*) across all replicates (Extended Data Fig. 1a). Violin plots of representative DEGs further demonstrate the enrichment of homeostatic genes in microglia and pro-inflammatory genes in monocytes (Fig. 2f, Supplementary Table 1).

## Brain engrafted monocytes exhibit a DAM-like signature

We were surprised to find that many of the genes enriched in monocyte engrafted mice (Fig. 2f) corresponded closely with the signature of ‘disease associated microglia (DAM)’, a transcriptional state that has consistently been observed in models of Alzheimer’s Disease (AD).^20^ In previous studies, we and others have similarly reported the induction of a DAM-like signature in human xMG adjacent to beta-amyloid plaques.^15,21,22^ In contrast to monocyte engrafted hFIRE mice, xMG engrafted mice in the current study , express low levels of DAM associated genes, and instead exhibit typical homeostatic microglial gene expression (Fig. 2d–f) and morphology (Fig. 1g,i,j). To further understand the potential similarity between the DAM signature and our spatial sequencing data, we used a previously published single cell sequencing (scSeq) dataset from xenografted AD mice^15,23^ to generate a DAM module score. Mapping of this module score across each of the 13 brain regions identified in our spatial analysis revealed a significant enrichment of the DAM signature across all regions of the monocyte engrafted brains without evidence of plaque or tangle pathologies in comparison to very low module scores within microglia-engrafted brains (Fig. 2g).

To determine whether our spatial sequencing analysis was consistent with the independently generated scSeq data presented in the accompanying study by Wang and colleagues, we compared the overlap in gene expression between both studies. This analysis revealed a highly significant overlap (P =1.98 × 10^−304^, Extended Data Fig. 1b) with over 40% of upregulated monocyte genes detected by Wang exhibiting a similar upregulation in monocytes within our spatial sequencing dataset. A Ratio-Ratio plot comparing Log2Fc expression differences between monocytes and microglia detected in both studies (Fig. 2h) likewise revealed a strong positive correlation (Pearson r=0.51, P < 2.2 × 10^−16^). To provide a third comparison between these two datasets we generated microglial and monocyte module scores from the Wang et. al. dataset and mapped those scores onto the 13 brain regions examined in our spatial sequencing data. This comparison again revealed a strong overlap between the two datasets across all 13 brain regions (Extended Data Fig. 1c). In summary, the spatial sequencing analysis performed in the current study and the scSeq analysis performed by Wang and colleagues reveal remarkably consistent and reproducible results, especially given the technical differences between the two studies which included differing sequencing approaches, donor cells and sources, mouse colonies, and ages of transplant.

## Proteomic analysis further distinguishes inflammatory monocytes from homeostatic microglia

**Figure 3.**
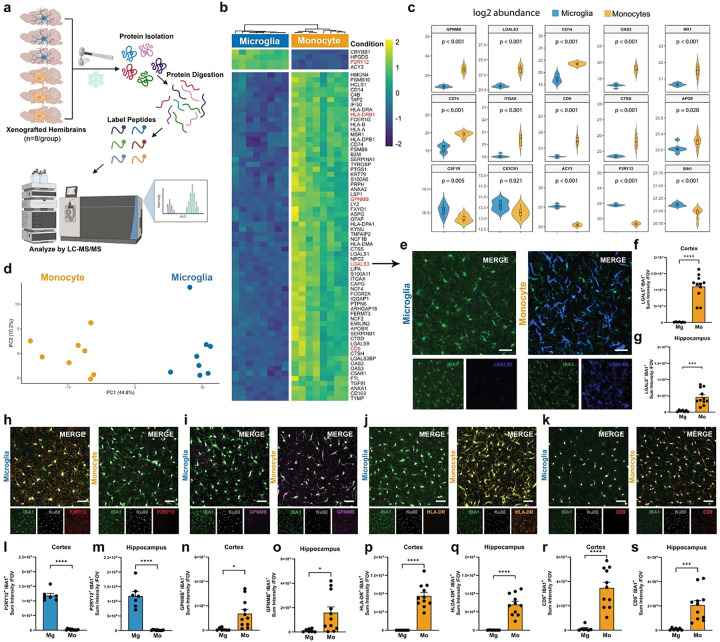
Proteomic analysis reveals increased levels of many proinflammatory proteins in monocyte engrafted brains. **(a)**Schematic depicting frozen hemibrain tissue and protein processing for TMT Mass Spec Analysis (n=8 mice per group, engrafted with n=4 Monocyte or Microglia lines). **(b)** Heatmap showing log2-transformed intensities of the top 70 differentially expressed proteins (DEPs) between microglia and monocytes engrafted brains (n=8 mice/group). **(c)** Expression levels of P2RY12, LGALS3, CD14, GPNMB, CX3CR1, CD74, ITGAX, OAS2, ACY3, CTSD, MX1, CD9, APOE, IFIT3, BIN1, and CSF1R protein. **(d)** Principal component analysis reveals microglia or monocyte identity as the primary source of variation between xenografted brains. **(e)** Representative immunostaining for LGALS3 positive (blue) microglia or monocytes (IBA1, green) in the cortex of xenografted mice; scale bar: 40μm. **(f-g)** Quantification of LGALS3 within the cortex and hippocampus, *p* values from unpaired, two-sided t test (Cortex t(16)= 7.568; ****P<0.0001) (Hippocampus: t(16)=4.571; ***P=0.0003). **(h-k)** Representative immunostaining for human nuclei (Ku80, white), P2RY12 (red), GPNMB (purple), HLA-DR (orange), and CD9 (pastel red) positive microglia or monocytes (IBA1, green) in the cortex of xenografted mice; scale bar: 40μm. Quantification of P2RY12 **(I,m)**, GPNMB **(n,o)**, HLA-DR **(p,q)**, and CD9 **(r,s)** immunoreactivity in the cortex and hippocampus of xenografted mice, *p* values from unpaired, two-sided t test (P2RY12 Cortex: t(16)= 20.83; ****P<0.0001) (P2RY12 Hippocampus t(16)= 9.605; ****P<0.0001) (GPNMB Cortex: t(16)= 2.904; *P=0.0104) (GPNMB Hippocampus t(16)= 2.424; *P=0.0276) (HLA-DR Cortex: t(16)= 7.592; ****P<0.0001) (HLA-DR Hippocampus t(16)= 6.299; ****P<0.0001) (CD9 Cortex: t(16)= 5.448 ****P<0.0001) (CD9 Hippocampus t(16)= 4.079; ***P=0.009). Data represented as average mean value ± SEM. Immunohistological analysis examined n=7 microglia and n=11 monocyte engrafted mice. ns, not significant; *p < 0.05, **p < 0.01, ***p < 0.001, and ****p < 0.0001.

In addition to our spatial sequencing analysis, rapidly frozen hemibrains from 8 mice per group were homogenized and examined by Tandem Mass Tag mass spectrometry (TMT-MS) (Fig. 3a). Our proteomic analysis revealed 1,406 differentially expressed proteins (DEPs; Adjusted P<0.05) between microglia and monocyte engrafted brains (Supplementary Table 2). Of those DEPs, 730 proteins were upregulated in monocyte engrafted brains whereas 676 downregulated DEPs were detected in monocyte engrafted mice (Extended Data Fig. 2a). However, when the top 70 DEPs were compared by heatmap, the great majority of DEGs were increased in monocyte brains, with only 4 out of 70 proteins exhibiting reduced expression in monocyte engrafted brains (Fig. 3b). Consistent with our spatial seq analysis, homoeostatic microglial proteins P2RY12, ACY3, and CRYBB1 exhibited increased expression in microglial engrafted brains, whereas monocyte engrafted brains exhibited greatly increased levels of proinflammatory myeloid and DAM-associated proteins including GPNMB, LGALS3 (Galectin 3), CD9, CD163, FTL (ferritin light chain), LIPA, ITGAX (CD11c) as well as Human leukocyte antigen (HLA) associated proteins involved in both MHC-I (HLA-A, HLA-B, B2M) and MHCII (HLA-DRA, HLA-DRB1, HLA-DPB1, CD74) antigen presentation (Fig. 3b). Violin plots of representative DEPs further demonstrate the consistent enrichment of myeloid activation-associated proteins in monocyte engrafted mice versus increased homeostatic proteins in microglia engrafted brains (Fig. 3c). Principle component analysis of our proteomics data demonstrated the consistency of these findings; revealing a clear separation between the 8 monocyte engrafted samples and 8 microglia engrafted samples along principal component 1 (PC1) representing 44.8% of the variance within the dataset (Fig. 3d).

Next, to further confirm that the expression of myeloid-associated DEPs in half brain homogenates was derived from engrafted human cells, we performed immunohistochemistry (IHC) for five representative DEPs along with IBA-1 to label all myeloid cells and the human-specific nuclear marker KU80 (Fig. 3e–s). IMARIS-based quantitative analysis revealed significantly increased expression of LGALS3 (Galectin-3) within engrafted monocytes in comparison to microglia within both the cortex and hippocampus (Fig. 3e–g). In contrast, IHC analysis of the homeostatic marker P2RY12 demonstrated far greater expression within microglia (Fig. 3h,I,m). Examination of three additional DAM-associated proteins (GPNMB, HLA-DRB1, CD9) also revealed greatly increased expression within human monocytes (Fig. 3 i–k,n–s).

To better define the functional pathways impacted by brain wide monocyte engraftment we performed Gene Ontology (GO) analysis of our proteomic dataset (Supplementary Table 3). Representative upregulated GO pathways included terms such as ‘Phagosome, antigen processing presentation, Lipid and atherosclerosis, efferocytosis, TNF signaling, JAK-STAT signaling, Ferroptosis, and cholesterol metabolism’ (Extended Data Fig. 3b). In contrast, GO pathways that were downregulated in monocyte engrafted mice highlighted terms such as Synaptic vesicle cycle, Cholinergic synapse, GABAergic synapse, Glutamatergic synapse, and Dopaminergic synapse (Extended Data Fig. 3c). Additional downregulated terms included Long-term potentiation (LTP), long-term depression (LTD), neurotrophin signaling, axon guidance, and longevity regulating pathways. As many of these downregulated pathways involve neuronal and synaptic proteins, we next sought to examine the potential impact of monocyte engraftment on non-myeloid cell types.

## Monocyte engraftment promotes the loss of neuronal, synaptic, and oligodendroglial proteins

**Figure 4.**
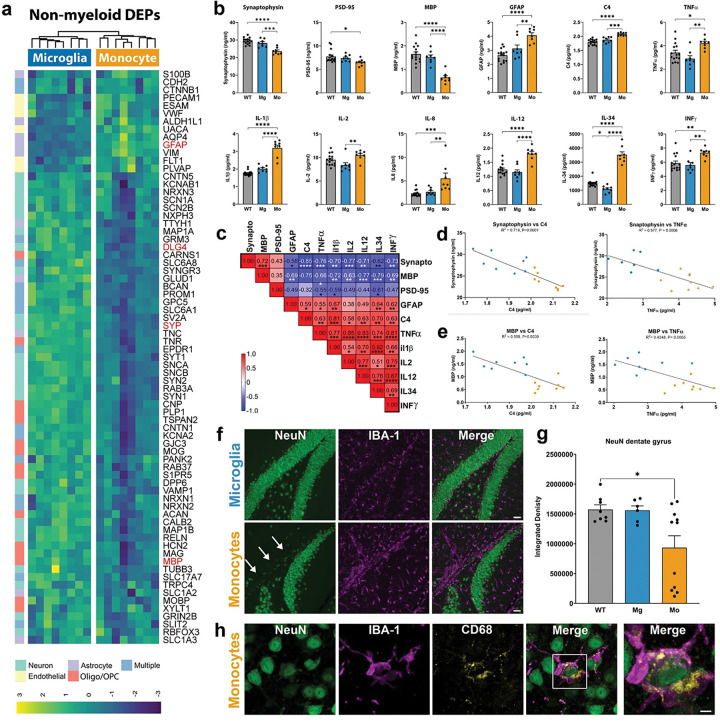
Proteomic analysis reveals consistent reductions in neuronal, synaptic, and oligodendroglial proteins in monocyte-engrafted mice. **(a)** Heatmap showing log2-transformed intensities in a subset of 74 non-myeloid DEPs between microglia and monocyte engrafted brains (n=8/group). **(b)** ELISA quantification of Synaptophysin (SYP), Post Synaptic Density-95 (PSD-95), Myelin Basic Protein (MBP), Glial Fibrillary Acidic Protein (GFAP), Complement component 4 (C4), Tumor Necrosis Factor-Alpha (TNF-α), Interleukin-1 beta (IL-1β), Interleukin-2 (IL-2), Interleukin-8 (IL-8), Interleukin-12 (IL-12), Interleukin-34 (IL-34), and Interferon gamma (IFN-γ) in soluble half-brain lysates; *p* values from one-way ANOVA (SYP: F(2,28)= 24.65; ****p<0.0001) (PSD-95: F(2,28)= 3.762; *p=0.0357) (MBP: F(2,28)= 27.82; ****p<0.0001) (GFAP: F(2,28)= 18.76; ****p<0.0001) (C4a: F(2,28)= 26.63; ****p<0.0001) (TNF-α: F(2,28)= 7.276; **p=0.0029) (IL-1β: F(2,28)= 77.13; ****p<0.0001) (IL-2: F(2,28)= 5.828; **p=0.0077) (IL-8: F(2,28)= 9.766; ***p=0.0006) (IL-12: F(2,28)= 22.34; ****p<0.0001) (IL-34: F(2,28)= 111.7; ****p<0.0001) (IFN-γ: F(2,28)= 7.455; **p=0.0025) with Tukey multiple comparisons tests. Data represented as average mean value ± SEM (WT, n = 15; Mg, n=8; Mo, n = 8). **(c)** Matrix of Pearson correlation (r) coefficients of quantified SYP, PSD-95, MBP, GFAP, C4, TNF-α, IL-1β, IL-2, IL-8, IL-12, IL-34, and IFN-γ. **(d-e)** Simple linear regression between levels of SYP and C4 (R^2^ = 0.719; ****P<0.0001), SYP and TNF-α (R^2^ = 0.577; ***P=0.0006), MBP and C4a (R^2^ = 0.558; ***P=0.0009), and MBP and TNF-α (R^2^ = 0.4348; **P=0.0055) in xenografted mice**. (f)** Representative confocal imaging of neuronal nuclei (NeuN, green) and xenografted microglia or monocytes (IBA1, green) in the ventral dentate gyrus, scale bar: 40μm. **(g)** Quantification of NeuN integrated density within the upper blade of the dentate gyrus of control (WT) or Monocyte (Mo) and Microglia (Mg) engrafted mice. As group distributions of NeuN deviated from normality, we utilized a non-parametric Kruskal–Wallis test (P=0.042) followed by Dunn’s multiple comparisons test, monocyte versus WT mice (Dunn’s: P=0.049), microglia versus WT mice (Dunn’s: P>0.999). **(h)** Representative confocal imaging of a neuronal nuclei (NeuN, green) surrounded by phagocytic (CD68, yellow) monocytes (IBA1, green) within the dentate gyrus, scale bar: 10μm. Data represented as average mean value ± SEM (WT, n = 8; Mg, n=6–7; Mo, n = 11). *p < 0.05, **p < 0.01, ***p < 0.0001, and ****p < 0.0001. Comparisons not shown are not significant.

While myeloid associated proteins were highly enriched in the top 70 upregulated DEPs (Figure 3b), our proteomic analysis further revealed a large number of non-myeloid DEPs that were significantly decreased in monocyte engrafted brains (Fig. 4a, Supplementary Table 2). Consistent with our analysis of down-regulated GO terms (Extended Data Fig. 2c), many of these non-myeloid DEPs represented canonical neuronal and synaptic proteins such as Tau (MAPT), β3-tubulin (TUBB3), Synaptophysin (SYP), Synapsin-1 (SYN1), Synaptic Vesicle Glycoprotein 2A (SV2A), and PSD-95 (DLG4). Likewise, we observed significant reductions in several canonical oligodendrocyte and oligodendrocyte precursor cell (OPC) proteins including Myelin Basic Protein (MBP), Myelin Oligodendrocyte Glycoprotein (MOG), Myelin-Associated Glycoprotein (MAG), 2’,3’-cyclic nucleotide 3’-phosphodiesterase (CNP), and proteolipid protein 1 (PLP-1) (Fig. 4a). To further confirm these key changes, we next measured levels of murine SYP, PSD-95, and MBP by ELISA. In each case we again observed a significant reduction in these proteins within monocyte engrafted brains (Fig. 4b), whereas microglia-engrafted mice showed no differences in comparison to ‘wild type’ hCSF1 immune-deficient controls.

## Monocytes increase astrogliosis and proinflammatory cytokines

While the majority of non-myeloid proteins exhibited decreased expression in monocyte-engrafted brains, several DEPs that are typically enriched in astrocytes or endothelial cells exhibited elevated expression (Fig. 4a, Supplementary Table 2). For example, Glial fibrillary acid protein (GFAP), a commonly utilized marker of reactive astrogliosis, along with the astrocytic proteins ALDH1L1, S100β, and aquaporin-4 (AQP4), exhibited elevated expression in monocyte brains (Fig. 4a). ELISA analysis of GFAP and Complement 4 (C4), both of which have frequently been associated with astrogliosis further corroborated our proteomic findings (Fig. 4b). Next, to determine whether the observed increases in myeloid cell activation were further accompanied by changes in cytokine production, we measured ten cytokines via multiplex ELISA. This analysis revealed significantly increased levels of six proinflammatory cytokines within monocyte engrafted brains including TNFα, Il1β, IL-2, IL-8, IL-12, and INFγ (Fig. 4b), whereas the four remaining analytes (IL-4, IL-6, IL-10, IL-13) showed no significant differences (Extended Data Fig. 3a). Interestingly, IL-34, which along with CSF1 signals via CSF1R to promote microglial differentiation and survival, was likewise elevated within monocyte engrafted brains. Importantly, none of these 10 cytokines were elevated within human microglial engrafted brains in comparison to wildtype hCSF1 immune-deficient controls. To further determine whether there might be a relationship between the observed reductions in synaptic and oligodendroglial proteins and increased proinflammatory cytokines we next generated a correlation table to compare each of the altered brain analytes. This comparison revealed highly significant correlations between many of these analytes including strong inverse associations (blue boxes) between synaptophysin and several proinflammatory cytokines (Fig. 4c, blue boxes). Individual regression analysis of synaptophysin versus C4 and synaptophysin versus TNFα further revealed highly significant correlations of R^2^=0.719 and R^2^=0.577 respectively (Fig 4d, P≤0.0006). These same two proinflammatory analytes also exhibited significant inverse relationships with MBP levels (Fig. 4e, P≤0.0055). Lastly, to determine whether the observed elevations in proinflammatory cytokines can also be detected in the periphery, we performed the same multiplex ELISA on serum samples collected from each mouse at the time of sacrifice. This analysis revealed significant increases in all ten analytes suggesting that monocyte engraftment within the brain also leads to a heightened proinflammatory environment within the periphery (Extended Data Fig. 3b).

## Human monocytes promote hippocampal neurodegeneration

Given that our proteomic analysis had detected significant decreases in neuronal proteins including Tau and β3-tubulin (Fig. 4a) and our spatial sequencing had revealed highly elevated levels of proinflammatory genes within the hippocampus (Fig. 2e, Extended Data Fig. 1), we performed IHC for the neuronal nuclear marker NeuN and examined the hippocampus. We were surprised to observe a dramatic loss of NeuN+ neurons within the upper blade of the dentate gyrus (Fig. 4f) in 5 of the 11 monocyte-engrafted brains examined. Because group distributions of NeuN deviated from normality, we utilized a non-parametric Kruskal–Wallis test followed by Dunn’s multiple comparisons, revealing a significant reduction in NeuN density in monocyte engrafted mice versus untransplanted ‘wildtype’ hCSF1 controls (Fig. 4g, Kruskal–Wallis: P=0.042, Dunn’s: P=0.049), but no significant differences between microglia engrafted mice and wildtype controls (Dunn’s: P>0.999). Interestingly, co-labeling with IBA-1 revealed densely packed ameboid-shaped monocytes in the same region of the upper blade where NeuN labeling had been lost in monocyte brains. We therefore wondered whether monocytes might be promoting the death and/or clearance of degenerating neurons within this region.

Monocytes, macrophages, and microglia can rapidly phagocytose dying cells. In fact, an important physiological function of monocyte-derived macrophages within the spleen is to phagocytose and degrade aging and dysfunctional red blood cells.^24^ However, phagocytosis proceeds rapidly, and antigenicity of internalized proteins is promptly lost by lysosomal degradation, making it challenging to reliably catch snapshots of cellular phagocytosis in the adult brain *in vivo*. Three recent reports have further demonstrated the difficulty of reliably quantifying microglial phagocytosis of cells, synapses, and myelin.^25–27^ Nevertheless, we observed many examples of NeuN+ neurons within the hippocampus that were surrounded by ameboid-shaped monocytes that expressed high levels of the phagolysosome marker CD68 (Fig. 4h), suggesting that monocytes rapidly clear or perhaps even promote the loss of neurons within the hippocampus. Examples of monocytes with internalized synaptophysin (Extended Data Fig. 4a,b) or myelin basic protein (Extended Data Fig. 4c,d) were also readily observed within the hippocampus and corpus callosum respectively.

While peptides can be rapidly cleared by the lysosome, a growing number of studies have demonstrated the accumulation and persistence of lipid droplets within microglia during neurodegenerative disease.^28–30^ Monocyte-derived macrophages within the periphery are likewise well recognized to accumulate lipid droplets during diseases such as arteriosclerosis, Non-Alcoholic Steatohepatitis, and lysosomal storage diseases.^31–33^ We therefore wondered whether brain engrafted monocytes might similarly exhibit lipid droplet accumulation. While IHC analysis of engrafted microglia revealed little to no accumulation of PLIN-2+ lipid droplets, engrafted monocytes exhibited a dramatic increase in lipid droplet density within the cortex and hippocampus (Extended Data Fig. 4e-h). Taken together, our findings suggest that peripherally derived human monocytes adopt a chronically pro-inflammatory signature that promotes the phagocytosis of neurons, synapses, and oligodendrocytes and the accumulation of lipid droplets. Importantly, the accompanying study by Wang and colleagues provides additional independent evidence that human myeloid cell ontogeny strongly influences neuronal and glial health and lipid droplet accumulation.

## The transcriptional signature of brain engrafted monocytes occurs in human neurodegenerative disease

CSF1R-related disorder (CSF1R-RD) is a rare neurodegenerative disease associated with mutations in CSF1R, a receptor that is critical for microglia differentiation, survival, and function.^34,35^ Also referred to as adult-onset leukoencephalopathy with axonal spheroids and pigmented glia (ALSP), patients affected by this disease exhibit decreased numbers of homeostatic microglia leading to demyelination, astrogliosis, and axonal spheroids which is accompanied by progressive cognitive and/or motor dysfunction.^36^ Recent studies have examined the potential use of bone marrow transplantation (BMT) to treat CSF1R-RD with highly conflicting results. For example, one study of 15 patients with a median follow-up of 26 months, found that BMT was associated with a significant worsening of cognitive symptoms.^37^ In contrast, another recent study reported that BMT slowed cognitive decline in five patients, although one of these five subjects exhibited Montreal Cognitive Assessment (MoCA) and mini mental state exam (MMSE) scores of 0–1 out of 30 throughout the study.

**Figure 5.**
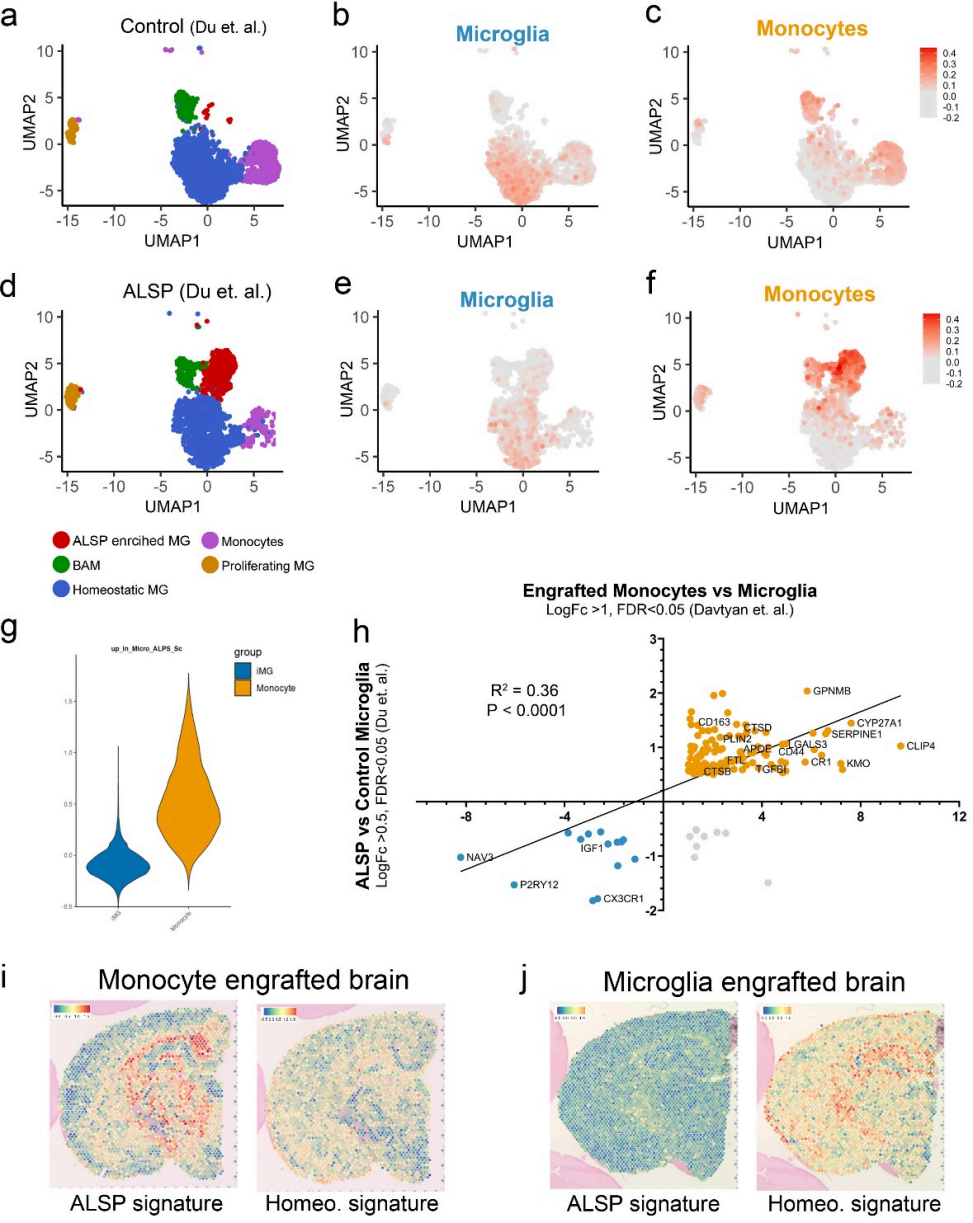
The transcriptional signature of brain engrafted monocytes can be detected in human neurodegenerative disease. **(a)** UMAP plot of homeostatic microglia (blue), proliferating microglia (yellow), ‘ALSP microglia’ (red), border associated macrophages (BAMs, green), and monocytes (purple) from control subjects (N = 10, n = 3,655 nuclei) and **(d)**patients with ALSP (N = 5, n=2,650 nuclei) reanalyzed from Du et. al., (Nature Immunology, 2025). The signature of xenografted microglia is enriched within the homeostatic microglia clusters of both control **(b)** and to a lesser extent ALSP **(e)** subjects. The xenografted monocyte signature is enriched within the monocyte and BAM populations of control subjects **(c)** but greatly increased in the ‘ALSP-microglia’ cluster of ALSP patients **(f)**. A module score calculated from Du et. al.’s ‘ALSP microglia’ signature is greatly enriched in monocyte engrafted mice **(g)**. Correlation analysis of log fold differences between ALSP and control microglia DEGs from Du et. al., in comparison to DEGs from spatial seq analysis of engrafted mice. Module scores generated from Du and colleagues’ ALSP-microglia or homeostatic-microglia signature mapped onto monocyte engrafted **(i)** or microglia engrafted **(j)** brain sections.

Most recently, an elegant single nuclei sequencing (snSeq) study compared the brains of five ALSP subjects to twelve control subjects.^38^ In addition to further demonstrating the loss of homeostatic microglia in ALSP brains, Du and colleagues detected a unique “ALSP-enriched microglia” signature that was virtually absent in controls. The authors further concluded that this unique cell cluster could either represent highly activated microglia or brain infiltrating “macrophages derived from peripheral monocytes”.^38^ Interestingly, DEG analysis of this ‘ALSP microglia’ cluster highlighted many genes that are also enriched in our current monocyte transcriptional signature, including *GPNMB, PLIN2, APOE, FTL, CD163, MITF, CHIT1, CR1, LGALS3, APOC1, ANXA2* among others (Fig. 2d,f; Supplementary Table 1). We therefore sought to directly compare our monocyte and microglia signatures to those observed in human control and ALSP brains by Du et. al. Using our spatial sequencing dataset we generated module scores from the top 200 genes upregulated in monocyte or microglia engrafted brains. These module scores were then mapped onto control and ALSP patient UMAPs that were reconstructed from Du et. al.’s snSeq dataset (Fig. 5a–f). Consistent with our previously published comparisons between brain-engrafted iPSC-microglia and human resection-derived *ex vivo* microglia,^15^ our microglial module score overlapped closely with homeostatic microglia identified by Du and colleagues (Fig. 5b,e). In contrast, our monocyte module score was greatly enriched within the unique cluster of ‘ALSP-microglia’ identified by Du et. al., (Fig. 5f).

To further compare these datasets, we performed the inverse analysis, calculating ‘control-microglia’ and ‘ALSP-microglia’ module scores from Du et. al., and demonstrating a strong enrichment of these modules in our microglia or monocyte engrafted brains respectively (Fig. 5g). In addition, we examined the log fold change in all significant myeloid DEGs detected by both studies. Regression analysis revealed a highly significant positive correlation (R^2^ = 0.36, P<0.0001), further demonstrating the correspondence between Du and colleagues ‘ALSP microglia’ and our current human monocyte engrafted samples. Lastly, we mapped the module scores calculated in Fig 5g onto our Visium spatial images, further demonstrating a strong enrichment of the ALSP-microglia module in our monocyte engrafted brains and a strong enrichment of the control homeostatic module in our iMG engrafted brains (Fig. 5i,j). Collectively, these analyses strongly suggest that the myeloid activation signature observed in our monocyte engrafted mice is closely related to a dysfunctional myeloid signature associated with a rapidly progressing and devastating human neurodegenerative disease.

## Monocyte engraftment promotes cognitive dysfunction

**Figure 6.**
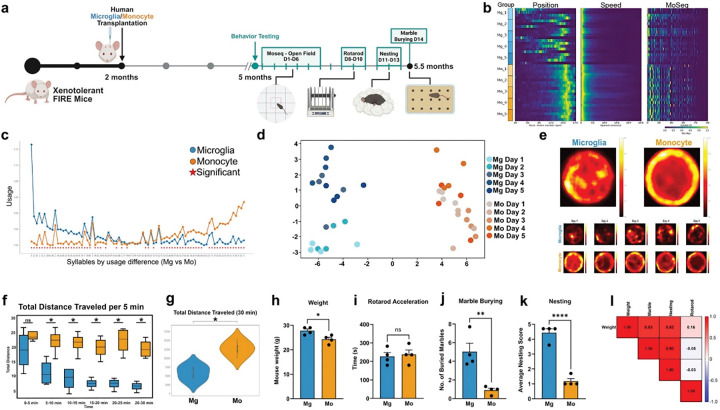
Brain wide engraftment of human monocytes promotes significant impairments in behavioral function. **(a)** Representative schematic for behavioral testing of xenografted hFIRE mice from 5 to 5.5-months of age. **(b)** Position, Speed, and Moseq syllable usage summary data for microglia (Mg) and monocyte (Mo) xenografted mice over 5 days (n=4 mice/group/day). **(c)** Moseq syllable usage of microglia (blue) or monocyte (orange) engrafted mice, stars represent differently used syllables between microglia mice and monocyte engrafted mice (see methods) **(d)** Linear Discriminant Analysis (LDA) of Mg or Mo xenografted hFIRE mice over 5 days of recording **(e)** Representative summary spatial maps of human microglia or monocyte xenografted mice averaged over five days of recording (top row, larger images) or across individual days (bottom row, small images). **(f)** Average total distance traveled per 5 min intervals of xenografted mice on day 1, Mann-Whitney-Wilcoxon test, *P<0.05. **(g)** Total distance traveled in open field arena over 30 min of recording, Mann-Whitney-Wilcoxon test *P<0.05 **(h)** Quantification of average weight of xenografted hFIRE mice at 5.5-months of age, p values from unpaired, two-sided t test (t(6)= 3.080; *P=0.0217). **(i)** Quantification of latency to fall off the accelerating rotarod over 5 min of testing, p values from unpaired, two-sided t test (t(6)= 0.3242; ns=0.7568). **(j)** Average number of marbles buried over 30 min of testing, p values from unpaired, two-sided t test (t(6)= 4.387; **P=0.0046). **(k)** Quantification of average nesting score over three days of recording, p values from unpaired, two-sided t test (t(6)= 9.971; ****P<0.0001). **(l)** Correlation matrix of behavioral testing of xenografted hFIRE mice. Data represented as average mean value ± SEM (Mg, n = 4; Mo, n = 4). *p < 0.05, **p < 0.01, and ****p < 0.0001. Comparisons not shown are not significant.

To determine the impact of the proinflammatory and hyperphagocytic profile of CNS-wide engrafted human monocytes on behavior, we quantified mouse exploratory behavior in the open field using Motion Sequencing (MoSeq), which uses machine learning to decompose movement into elemental motifs of action (e.g., runs, rears, turns) called “syllables”.^39^ We quantified the expression of individual behavioral syllables expressed by transplanted mice at 5 months of age in an open-field arena over five consecutive days. We then subsequently performed three additional behavioral assays: rotarod, nesting, and marble burying (Fig 6a). In addition to revealing patterns of syllable usage, the MoSeq framework provides quantitative summary data r that revealed a striking difference in the position of mice transplanted with microglia versus monocytes as well as readily apparent difference in syllable usage (Fig. 6b). Consistent with pervasive behavioral differences between monocyte and microglia-transplanted mice, 78% of all syllables identified exhibited significant differences in usage in the open field (Fig 6c). Linear discriminant analysis (LDA) revealed a clear separation in the overall patten of syllable use between the transplanted mice that was stable over days (Fig. 6d). Visualization of the arena over 30 mins also revealed pronounced thigmotaxis within the monocyte engrafted mice as they paced along the arena walls (Fig. 6e). During the first minute of observation both monocyte and microglia mice exhibited typical murine behavior, exploring the border of the arena. However, over 30 minutes, monocyte engrafted mice continued to exhibit hyperactivity, showing no signs of habituation and leading to a significant increase in total distance traveled over 30 minutes (Fig. 6f,g).

Sickness behavior in mice includes lethargy and reduced appetite and can be induced by proinflammatory stimuli such as lipopolysaccharide (LPS).^40^ Similar to LPS, we found that monocyte engraftment leads to increased levels of multiple proinflammatory cytokines within both the brain and periphery (Fig. 4b, Extended Data Fig 3b). We therefore measured the weight of mice prior to sacrifice, detecting a significant reduction in the weight of monocyte engrafted mice (Fig. 6h). Despite this, monocyte and microglia engrafted mice exhibited equivalent motor function via accelerating Rotarod (Fig. 6i). However, in two other naturalistic tasks: marble burying and nest-building, monocyte engrafted mice exhibited greatly reduced activity (Fig. 6j,k). Low levels of nest building and burying activity have previously been shown to provide a sensitive measure of health and depression-like states and can be induced by systemic LPS treatment.^41,42^ Therefore, to determine whether the observed behavioral impairments might be associated with reductions in weight, we performed a correlation analysis. This comparison revealed strong positive correlations between weight and both nest building (r=0.82, *P<0.013) and marble burying (r=0.83, *P<0.012), suggesting that the observed reductions in weight and naturalistic behavior in monocyte engrafted mice provide evidence of sickness behavior. Collectively, our behavioral analyses strongly indicate that widespread engraftment of human monocytes leads to significant neurobehavioral dysfunction.

## Discussion

Microglia arise early in development from HPCs within the yolk sac that give rise to primitive macrophages that migrate into the developing CNS.^11,12^ In contrast, blood monocytes and most other peripheral myeloid cells arise later in development and continue to be produced throughout life from definitive bone marrow-derived HSPCs.^11,12^ Lineage tracing studies have further confirmed that the CNS receives little-to-no input from definitive HSPC-derived monocytes or macrophages in healthy mice.^10^ Nevertheless, recent studies have coupled transplantation of definitive HSPCs, myeloid progenitors, or monocytes with microglia depleting treatments, to replace endogenous microglia with peripherally derived murine myeloid cells.^1–5^ These initial studies have shown exciting evidence of reduced pathology and improved outcomes in mouse models of AD, LSD, and FTD. However, these along with other studies have also shown that engrafted MLCs remain transcriptionally and functionally distinct from microglia.^6–9^ As human and murine microglia also exhibit many species-dependent differences,^8,13–16^ the potential impact of human MLCs on brain function remains unclear. It is therefore critically important to investigate the effects of human MLC engraftment in appropriate *in vivo* systems before proceeding to clinical translation.

The current study together with the accompanying report by Wang et. al., independently investigated the differences between human iPSC-microglia and peripherally derived monocytes following transplantation into the brains of adult microglia-deficient xenotolerant FIRE mice. Four months post-transplantation, despite similar numbers of engrafted cells and widespread brain distribution, we detected many important differences in the transcriptional and functional properties of brain engrafted monocytes versus microglia. Specifically, we find that human monocytes, but not microglia, led to a chronic proinflammatory state characterized by pronounced astrogliosis, demyelination, and neuronal and synaptic loss, culminating in severe behavioral impairments. Collectively, our data demonstrate that ontogeny plays a critical role in the long-term function of myeloid cells within the brain, suggesting that clinical development of approaches that promote the replacement of microglia with definitive myeloid cells instead of primitive-like iPSC-microglia warrants considerable caution.

A major strength of our findings is the remarkable similarities between our current results and the accompanying report by Wang and colleagues. Each study was conceived, pursued, and interpreted independently and yet comparisons between the two datasets reveal extremely strong concordance (Fig. 2h, Extended Data Fig. 1b,c). Importantly, the two studies also utilized complimentary approaches to examine the impact of myeloid cell ontogeny on brain health. Whereas Wang et.al., utilized epigenetic approaches to further understand the transcriptional regulation of definitive versus primitive myeloid ells, we utilized proteomic and behavioral approaches to further examine the downstream consequences of these differing ontogenies.

Despite these robust and consistent results, it is important to acknowledge some potential limitations of our study. To achieve brain wide engraftment of human monocytes in a model system we utilized immune deficient mice that lack, B, and NK cells. As a result, potential interactions between adaptive immunity and innate myeloid cells within the brain cannot be examined. The future development of more complex humanized models that employ matching definitive and primitive immune cell engraftment could provide a promising approach to address this shortcoming. Another potential caveat to our approach is that we examined the differences between complete brain wide monocyte versus microglia engraftment. Clinical approaches would be unlikely to fully replace endogenous microglia and thus understanding the potential interactions between engrafted monocytes and endogenous microglia will likely be informative. For example, can proinflammatory monocytes alter the activation state of endogenous microglia or vice versa? Future studies that examine co-transplantation of human microglia and monocytes could therefore be highly informative.

In conclusion, the current study demonstrates the importance of human myeloid cell ontogeny on neuronal and glial health and behavioral function. Together with the accompanying study by Wang et. al., our findings suggest that CNS wide replacement of microglia with definitive human myeloid cells should be examined with considerable caution and that the use of stem cell derived microglia that better mimic the primitive ontogeny of microglia may provide a safer alternative.

## METHODS

### Animals

All animal procedures were conducted in accordance with guidelines set forth by the National Institutes of Health and the University of California, Irvine Institutional Animal Care and Use Committee. Original CSF1R^ΔFIRE/ΔFIRE^ mice were generated and previously characterized by Clare Pridans and David Hume.^43^ FIRE mice were generated on a B6CBAF1/J background. Heterozygous FIRE mice were then crossed with wild-type C57BL/6J mice for four generations before backcrossing for four generations with xenotransplantation-compatible MITRG mice (Jackson Laboratories # 017711) which harbor deletions in Rag2 and IL2rγ and humanized CSF1, CSF2/Il3 and THPO. The resulting mice were maintained via breeding of heterozygous FIRE females (Fire^−/+^, Rag2^−/−^, il2rg^−/−^, hCSF1^+/+^, hCSF2/Il3^+/+^, hTHPO^+/+^) with homozygous (Fire^−/−^, Rag2^−/−^, il2rg^−/y^, hCSF1^+/+^, hCSF2/Il3^+/+^, hTHPO^+/+^) or heterozygous males (Fire^−/+^, Rag2^−/−^, il2rg^−/y^, hCSF1^+/+^, hCSF2/Il3^+/+^, hTHPO^+/+^). Genotyping of FIRE mice was performed using the following primers: mCSF1R_FIRE_F (5’-GCGGTTGTAGGAAACCCTGA-3’), mCSF1R_WT_F (5’-GGTGCCAGCAATGTGTTTCC-3’), and mCSF1R_R: (5’-CACTCCTACCACTGGGCATC-3’). All mice were age- and sex-matched and group-housed, but single-housed post-transplantation, on a 12-h/12-h light/dark cycle with food and water *ad libitum*. Mice were housed with ambient temperature and humidity. Cages and bedding were changed every 1–2 w. Behavioral analysis utilized hFIRE mice,^19^ a highly similar xenotolerant model that lacks murine microglia but does not include humanized CSF2 and THPO.

### Cell lines

Peripheral Blood negatively sorted CD14^+^ monocytes from four male subjects were obtained from Lonza (#4W-400; see details below). Four iPSC lines from healthy male subjects were generated and obtained from the Alzheimer’s Disease Research Core (ADRC) at University of California Irvine (see details in the table). Informed consent was received under an approved institutional IRB and Non-integrating Sendai virus (Life Technology, Cytotune 2.0) used to perform iPSC reprogramming. iPSCs clones were then confirmed to exhibit normal karyotype by Array Comparative Genomic Hybridization (ACGh by Cell Line Genomics), exhibit pluripotency via trilineage in vitro differentiation (Trilineage Differentiation Kit, Stem Cell Technologies), and sterility via MycoAlert (Lonza).

**Table:** 

Sample	Cell Type	Line ID	Sex	Age (at donation)	Race	Source
1	CD14+ mono	26198	Male	23	Caucasian	Lonza
2	CD14+ mono	23617	Male	45	Caucasian	Lonza
3	CD14+ mono	23623	Male	29	Unknown	Lonza
4	CD14+ mono	29631	Male	49	Caucasian	Lonza
5	iPSC	ADRC 75	Male	64	Caucasian	UCI ADRC
6	iPSC	ADRC 76	Male	83	Caucasian	UCI ADRC
7	iPSC	ADRC 77	Male	77	Caucasian	UCI ADRC
8	iPSC	ADRC 40	Male	66	Asian	UCI ADRC

### Microglia differentiation from iPSCs

iPSC-derived microglia (iMG) were differentiated using our previously published and highly replicated protocol.^44^ To begin HPC differentiation, iPSCs were passaged onto 6-well matrigel-coated plates in TeSR-E8 at a density of 80 colonies of 100 cells each per 35 mm well. On day 0, cells were transferred to Medium A from the STEMdiff Hematopoietic Kit (05310; STEMCELL Technologies). On day 3, cells were exposed to Medium B and remained in Medium B for 7 additional days while small round HPCs began to lift off the colonies. On day 10, non-adherent CD43+ HPCs were collected by carefully removing medium and non-adherent cells with a serological pipette. At this point, HPCs were frozen in BamBanker freezing solution (Wako, NC9582225) for long-term storage. For iMG differentiation, HPCs were cultured on 6-well matrigel-coated plates in complete iMG medium (DMEM/F12 [11039–021; Thermo Fisher Scientific], 2× insulin-transferrin-selenite [41400045; Thermo Fisher Scientific], 2× B27 [17504044; Thermo Fisher Scientific], 0.5× N2 [17502048; Thermo Fisher Scientific], 1× Glutamax [35050–061; Thermo Fisher Scientific], 1× non-essential amino acids [11140–050; Thermo Fisher Scientific], 400 mM monothioglycerol, and 5 mg/mL human insulin [I2643; Sigma-Aldrich] freshly supplemented with 100 ng/mL IL-34 [200–34; Peprotech], 50 ng/mL TGFb1 [100–21; Peprotech], and 25 ng/mL M-CSF [300–25; Peprotech]) for 28 days. Fresh complete iMG media was added every 24 h. iMG were frozen in BamBanker freezing solution (Wako, NC9582225) for long-term storage. Cells used for iMG transplantation were later thawed in complete iMG medium for 18–24 h to recover before being resuspended at 62,500 cells/μl in 1× Dulbecco’s Phosphate Buffered Saline (DPBS; low Ca2+, low Mg2+) for transplantation. A total of 500K cells (Monocyte or iMG) were injected per mouse.

### Adult Intracranial Transplantation

All mouse surgeries and use were performed in strict accordance with approved National Institutes of Health (NIH) and institutional guidelines. Direct bilateral intracranial injections were performed as previously described.^15,45^ Briefly, adult mice (2 m old) were anesthetized under continuous isoflurane and secured to a stereotaxic frame (Kopf), and local anesthetic (Lidocaine 2%; 17478–711-31, Medline) was applied to the head before exposing the skull. Using a 30-gauge needle affixed to a 10-mL Hamilton syringe, mice received 2 μL of cell suspension in sterile 1× DPBS (14190144; Thermo Fisher Scientific) at 125,000 cells/μL at each injection site. Monocyte and iMG transplantations were conducted bilaterally into the lateral parietal association cortex and dorsal hippocampus at the following coordinates relative to bregma: anteroposterior, 2.06 mm; mediolateral, ± 1.75 mm; dorsoventral, 1.75 mm (hippocampus) and 0.95 mm (cortex). Cells were injected at a rate of 62,500 cells/30 s with 4 min diffusion time in between injections. The needle was cleaned with consecutive washes of DPBS, 70% (vol/vol) ethanol, and DPBS in between hemispheres and animals. Animals were allowed to recover on heating pads before being placed in their home cages and received 2 mg/mL Acetaminophen (0904–7014-16; Major Pharmaceuticals) diluted in water for 5 d.

### Collection and Isolation of Peripheral Blood Plasma

Blood was collected into an EDTA-anticoagulant collection tube and immediately placed on ice. Tubes were then transferred to a pre-chilled centrifuge and spun at 1,600 rcf for 15 min at 4°C. Following centrifugation, plasma supernatant was gently collected and transferred to a 1.5 mL Eppendorf tube on ice. Eppendorf tubes were then transferred to a pre-chilled centrifuge and spun at 1,600 rcf for 15 min at 4°C. Following the second centrifugation, plasma supernatant was collected and aliquoted into 30 μL sub-aliquots and kept at −80°C for long-term storage.

### Isolation of Soluble Protein Homogenates

Animals were perfused (20 mL per min) with 1X DPBS (4°C) for 3 min. Brains were surgically excised from each mouse and cut in half along the mid-sagittal plane. The left hemisphere was placed in 4% (wt/vol) paraformaldehyde (PFA) for 36 h at 4°C for subsequent immunohistochemistry (IHC) and spatial transcriptomics and the right hemisphere fresh-frozen on dry ice and stored at −80°C for biochemical analysis and proteomics. Fresh frozen hemispheres were crushed on liquid nitrogen using mortar and pestle and were split into 2 aliquots. One aliquot was used for Proteomics. The second aliquot was homogenized in solution of T-PER (78510, Thermo Scientific, Waltham, MA) and phosphatase and protease inhibitor mixtures (78426 and 78426, Thermo Scientific, MA). Homogenates were centrifuged at 16,000 rcf for 30 min at 4°C and supernatants were stored at −80°C for analysis. Protein concentration of soluble brain samples was determined using BCA Protein Assay Kit (Pierce) and 2 mg/mL of total protein per sample was run for ELISAs.

### Quantitative Biochemical Analysis

Analysis of soluble brain samples were conducted using commercially available ELISAs following manufacturer guidelines to measure Glial Fibrillary Acidic Protein (ABIN6574131; antibodies-online.com), Synaptophysin (ABIN6959799; antibodies-online.com), Postsynaptic density protein 95 (K15OQND; MSD), human XRCC5/Ku80 (MBS7608104; MyBioSource.com); human complement C4 (ab108824; Abcam); mouse IL-34 (M3400; R&D systems) and V-PLEX Proinflammatory Panel 1 Human Kit (K15049D; MSD). Analysis of peripheral blood plasma was also conducted using the V-PLEX Proinflammatory Panel 1 Human Kit (K15049D; MSD). Protein levels for each sample were then calculated via comparison to each assay-specific standard curve.

### Immunohistochemistry

Animals were perfused with DPBS and isolated brain hemispheres were drop fixed in 4% (wt/vol) PFA for 36 h then cryoprotected in a 30% (wt/vol) sucrose at 4°C. Brains were sectioned coronally into 30-μm-thick slices on a freezing microtome (Leica, SM 2010R) and stored in a solution of 0.05% NaN3 (S2002; Sigma-Aldrich) in 1× PBS (P44017–100TAB; Sigma-Aldrich) as free-floating slices. For staining, tissue was blocked for 1 h in 1× PBS, 0.2% Triton X-100 (9002–93-1; Thermo Fisher Scientific), and 10% donkey serum (NC9624464; Thermo Fisher Scientific). Immediately following blocking, brain sections were placed in primary antibodies diluted in 1× PBS and 1% donkey serum and incubated ON whilst shaking at 4°C. Samples were then incubated in conjugated secondary antibodies for 1 h followed by mounting on microscope slides. Sections were labeled with combinations of goat anti-IBA1 (1:300; ab5076; Abcam), rabbit anti-IBA1 (1:300; 019–19741; Wako), rabbit anti-Ku80 (1:200; ab80592; Abcam), mouse anti-Ku80 (1:250; MAS-12933; Invitrogen), rabbit anti-P2RY12 (1:500; HPA014518; Millipore Sigma), and chicken anti-GFAP (1:2000; ab4674; Abcam) overnight at 4°C followed by 1 h incubation at 23°C whilst shaking. After washing with 1× PBS for 5 m three times, sections were incubated with highly cross-adsorbed AlexaFluor-conjugated secondary antibodies (1:400; ThermoFischer) for 1 h in the dark, then washed three times with 1× PBS before mounting with Fluoromount-G (0100–01; SouthernBiotech).

### Imaging acquisition and processing

Immunofluorescent sections were visualized and captured using an Olympus FX3000 confocal microscope using identical confocal and Z-stack settings for a given quantitative comparison. Half brain stitches to capture half-brains with high resolution were performed using Fluoview FV31S-DT software. For each quantitative analysis, three sections were imaged per region per animal using the “Surfaces” function in IMARIS software (Bitplane). All images were examined blinded using batch processing. Numbers, area, volume, or intensity were calculated for each section, and these values were averaged to provide a single value per animal for subsequent statistical comparisons. For some representative images, brightness and contrast settings were slightly and equally adjusted across groups to reveal fine structures and morphology. Importantly, no changes were made to any images used for quantification.

### Spatial transcriptomics and analysis

Fixed half brains were cryoprotected in 30% sucrose and then sectioned in the coronal plane with a freezing sliding microtome. 10μm thick sections at the level of the dorsal hippocampus (Bregma AP −2.06) were collected and mounted on Superfrost plus glass microscope slides and stored at 4°C overnight to dry. Slide preparation was performed following the 10X Visium CytAssist Spatial protocol (Rev A). Each slide contained two capture areas, and each capture area was designed to hold three adjacent half brain coronal sections, allowing for parallel spatial profiling of multiple experimental conditions within a single assay. Sections were stained with hematoxylin and eosin (H&E) and imaged using brightfield microscopy to capture histological architecture. Spatial transcriptomic libraries were prepared using the 10x Genomics Human Transcriptome Probe Set v2, which uses probe hybridization and ligation-based capture (rather than poly-A enrichment), making it suitable for RNA derived from Paraformaldehyde fixed tissue. Although this probe set is designed to detect human transcripts, expression of genes such as GFAP, AQP4, and SNAP25—typically associated with murine astrocytes and neurons—was also observed. These signals were not computationally removed. Instead, it was assumed that myeloid-associated transcripts were derived from the engrafted human cells, as the host FIRE mouse lacks murine microglia. Library preparation, probe hybridization, and amplification were carried out by the UCI Genomics Core Facility, following the manufacturer’s protocols. Sequencing was performed on an Illumina NovaSeq 6000 platform using paired-end reads (28 bp for read 1 and 91 bp for read 2), achieving a target sequencing depth of 50,000–100,000 reads per spot, consistent with recommended parameters for FFPE tissue. Each Visium spot represents pseudobulk gene expression from approximately 1 to 10 cells, depending on local tissue density and spot coverage.

### Tissue Demultiplexing and Brain Region Annotation

Each capture area included three distinct brain tissue sections, representing either biological replicates or different experimental conditions. To demultiplex the spatial data, manual segmentation was performed in Loupe Browser v8.0 or higher (10x Genomics). Using the aligned H&E-stained image, each tissue section was visually outlined based on its physical boundaries, and only barcodes falling entirely within each outlined area were extracted for downstream analysis. There was no spatial overlap between the three brain tissue sections within each capture area, ensuring that each barcode could be unambiguously assigned to a single tissue section. Spots located outside the H&E-detected tissue area were excluded from the analysis. To annotate anatomical brain structures, each tissue section was manually assigned to one of 13 predefined brain regions, including upper and lower cortical layers, the hippocampus, striatum, thalamus, amygdala, and white matter tracts (Fig 2a). These assignments were guided by visual alignment to reference coronal sections from the Allen Mouse Brain Atlas, using anatomical landmarks such as ventricles, white matter tracts, and cortical boundaries. The resulting barcode-to-region assignments were added to the Seurat object as metadata and were used in region-stratified analyses, including differential gene expression, module score comparisons, and spatial visualization.

### Data Preprocessing and Quality Control

Sequencing reads were processed using Space Ranger v3.1.0 with the appropriate GRCh38 reference genome and FFPE Human Transcriptome Probe Set v2 annotation files (10x Genomics). Raw outputs—including feature-barcode matrices, spatial image data, and tissue position files—were imported into R (v4.4.2) using the Load10X_Spatial() function from Seurat v5.3.0. Given the fragmented nature of RNA in FFPE tissue, a rigorous quality control (QC) pipeline was applied to retain only high-quality barcodes corresponding to biologically informative tissue spots. Global distributions of key QC metrics were visualized using observed quantile plots, allowing empirical determination of filtering thresholds. The following filters were applied: Total UMI count (nCount_Spatial) > 800, Number of detected genes (nFeature_Spatial) > 300, Percentage of mitochondrial transcripts < 10%, Complexity score > 0.05. The complexity score was calculated as the ratio of unique genes to total UMI per spot: complexity = nFeature_Spatial / nCount_Spatial. This metric captures transcript diversity relative to sequencing depth and helped identify and exclude low-complexity (potentially damaged or ambient RNA–dominated) barcodes. Barcodes outside the tissue area, as defined by H&E-based segmentation, were excluded during demultiplexing. Each spot was annotated with sample ID, experimental condition, slide ID, and capture area as metadata. Slide- and region-specific barcode prefixes were standardized to enable consistent downstream merging and integration.

### Normalization, Integration, and Dimensionality Reduction

Each sample was independently normalized using SCTransform, which stabilizes variance while adjusting for technical confounders such as sequencing depth and mitochondrial gene content. To account for batch effects due to differences in slide, capture area, and processing time, data were integrated using Harmony, which aligns datasets in principal component (PC) space. Slide ID and capture area were specified as covariates for batch correction, enabling alignment of biological signals across technical replicates while preserving spatial and anatomical structure. Dimensionality reduction was performed using Principal Component Analysis (PCA) followed by Uniform Manifold Approximation and Projection (UMAP) for two-dimensional visualization. Graph-based clustering was performed using Seurat’s FindNeighbors() and FindClusters() functions with resolutions ranging from 0.4 to 0.8. Clusters were assessed for biological granularity and stability across resolutions. A resolution of 0.6 was selected for downstream analyses, as it produced a non-redundant but sufficiently detailed set of clusters. This choice was informed by cluster tree analysis and best practices outlined in the Bioconductor OSCA workflow (https://bioconductor.org/books/3.12/OSCA/clustering.html). Spatial feature plots were generated using Seurat functions and maintained precise registration to H&E histological images, preserving anatomical context for downstream spatial analyses.

### Differential Gene Expression Analysis

To quantify transcriptional differences across conditions and regions, we applied two complementary frameworks—spot-level testing and pseudobulk aggregation—to retain spatial resolution while adding replicate-aware statistical control to limit false positives. Spot-level differential expression was performed on SCT-normalized data using Seurat’s FindMarkers() (Wilcoxon rank-sum), including batch covariates in the model. Significance was defined as Benjamini–Hochberg–adjusted P (FDR) < 0.05 and |log_2_FC| > 1. This approach is sensitive to spatially localized or cell-type–specific differences but treats spots as independent replicates, which can inflate false discoveries due to within-sample correlation—a recognized limitation in single-cell/spatial analyses.^46,47^ Pseudobulk differential expression was also performed to incorporate biological replication and mitigate pseudo-replication, raw counts were aggregated per sample and analyzed with edgeR v3.42.4 by contrasting Monocyte condition to iMG. Libraries were normalized using TMM, and GLMs were fit with condition and batch covariates. Genes were called significant at FDR < 0.05 and |log_2_FC| > 1. This replicate-aware framework improves control of false discovery and detects global, consistent shifts across samples. Combining both spot-level and pseudobulk frameworks balances spatial sensitivity (spot-level) with statistical robustness (pseudobulk). We prioritized high-confidence DEGs detected by both methods; spot-only DEGs were interpreted as localized/cell-type–restricted signals representing spatially resolved transcriptional programs, whereas pseudobulk-only DEGs reflected broad but modest sample-level/condition specific shifts. This dual strategy follows best-practice recommendations for spatial/single-cell DE.^46^

### Module Score Calculation

Gene program activity per spatial transcriptomics spot was quantified using Seurat’s *AddModuleScore()* function, applied to SCT-normalized expression values. We curated three transcriptional modules to capture distinct myeloid activation states relevant to the xenotransplant context: 1) Disease-Associated Microglia (DAM), 2) Homeostatic microglia (*P2RY12, P2RY13, CX3CR1, ACY3*, *MX1*) and 3) Monocytes (*CD14, MSR1, LGALS1, GPNMB, ITGAX*). Gene sets were manually curated based on prior literature and pathway relevance.

### Spatial-seq versus RNA-seq Conmparisons

Genes were plotted in a ratio–ratio plot and points were colored according to significance and directionality thresholds. Overall concordance between datasets was quantified using the Pearson correlation coefficient. Venn plots comparing upregulated monocyte genes across the two studies compared genes that reached significance in both studies (Log2FC>1, FDR<0.05) via hypergeometric P.

### Visualization and Statistical Reporting

#### Intersection (Venn) analysis :

Overlap between spot-level and pseudobulk DEGs was summarized with VennDiagram with *ggven* reporting counts and percentages for each set and the intersection. Genes in the intersection were prioritized as high-confidence significant results for downstream summaries and figures. *Volcano plots*: Differential expression results were visualized with *EnhancedVolcano* using the pseudobulk result as background, displaying log_2_FC vs. −log_10_(FDR). The significant genes (intersection of the two methods) were colored coded by group (iMG vs. Monocyte), with selected marker genes labeled using *ggrepel* to avoid overlap.

#### Violin plots with statistics:

Expression of selected genes and module scores was summarized with violin plots (ggplot2), showing median and interquartile ranges. Two-sided Wilcoxon rank-sum tests were performed using *ggpubr::stat_compare_means*, with Benjamini–Hochberg correction applied when testing multiple genes/regions. Adjusted P values were displayed directly on the plots. Spatial maps genes and modules: Spatial distributions of single genes and module scores (DAM, IFN, iMG, Monocyte) were rendered with Seurat’s *SpatialFeaturePlot* and *SpatialDimPlot*, and, where helpful, customized using *SeuratExtend/scCustomize* for consistent legends and image ordering across samples. All spatial plots were registered to H&E images.

### Proteomics

Tandem mass tag (TMT)–labeled brain tissue lysates was processed as previously described.^48,49^ Samples (100ug) were reduced and alkylated with 5 mM DTT and 10mM IAA in the dark for 30 min. The samples were then digested with 4ug of Lysyl endopeptidase (Wako) overnight. Samples were then diluted with 7X 100mM Tris with 4ug trypsin (ThermoFisher) and digested overnight at room temperature. The peptide solutions were acidified to a final concentration of 1% (vol/vol) formic acid (FA) and 0.1% (vol/vol) trifluoroacetic acid (TFA) then mixed 1:1 with 4% H3PO4 and desalted with a 10 mg Prime MXC plate (Water’s). Each MCX column was loaded directly after acidification. The wells were then washed with 500ul of L 100 mM NH4COOH / 2% formic acid and then with 500ul of methanol. Samples were eluted with 500ul of (50:50 ACN:MeOH + 5% NH4OH) and immediately dried by a SpeedVac. For TMT labeling, each brain peptide digest was resuspended in 75 μL of 100 mM triethylammonium bicarbonate (TEAB) buffer, and 5 mg of TMTpro reagents was dissolved in 200 μL ACN. Then 100 μg of peptide samples were aliquoted and resuspended in 100 μL TEAB buffer. After bringing the TMTpro reagents to room temperature and mixing with ACN, 41 μL of the TMT solution was added to each peptide solution and incubated for 1 h. The reaction was stopped with the addition of 8 μL of 5% hydroxylamine. The labeled samples were then combined, concentrated using a SpeedVac, and diluted with 0.1% TFA. After acidification, the peptides were desalted using a C18 Sep-Pak column, washed, and eluted with 50% ACN. Each sample was assessed for complete labeling on a single-shot LC-MS/MS analysis prior high pH fractionation.

### High-pH Off-line Fractionation

Dried samples were re-suspended in high pH loading buffer (0.07% vol/vol NH4OH, 0.045% vol/vol FA, 2% vol/vol ACN) and loaded onto a Water’s BEH 1.7 um 2.1mm by 150mm. A Thermo Vanquish was used to carry out the fractionation. Solvent A consisted of 0.0175% (vol/vol) NH4OH, 0.01125% (vol/vol) FA, and 2% (vol/vol) ACN; solvent B consisted of 0.0175% (vol/vol) NH4OH, 0.01125% (vol/vol) FA, and 90% (vol/vol) ACN. The sample elution was performed over a 25 min gradient with a flow rate of 0.6 mL/min. A total of 192 individual equal volume fractions were collected across the gradient and subsequently pooled by concatenation into 96 fractions and dried to completeness using a SpeedVac.

### Liquid Chromatography

Each sample was resuspended in 10 ul of loading buffer (0.1% FA) and 2 ul was analyzed by liquid chromatography coupled to tandem mass spectrometry. Peptide eluents were separated on Water’s CSH 150um x 15cm column packed with 1.7um resin. Buffer A was water with 0.1% (vol/vol) formic acid, and buffer B was 80% (vol/vol) acetonitrile in water with 0.1% (vol/vol) formic acid. Elution was performed over a 30 min gradient. The non-linear gradient was from 1% to 99% solvent B.

### Mass Spectrometry

Peptides were monitored by Orbitrap Eclipse spectrometer (ThermoFisher) fitted with a high-field asymmetric waveform ion mobility spectrometry (FAIMS Pro) ion mobility source (ThermoFisher). Two compensation voltages (CV) of −45 and −65 were chosen for FAIMS. Each 1.5 second cycle consisted of one full scan (MS1) was performed with an m/z range of 410–1600 at 60,000 resolution, 100% AGC and 50 ms injection time and higher energy collision-induced dissociation (HCD) TMT scans collected at 30,000 resolutions with Turbo TMT on. The mass range was set to start at 120 m/z, isolation was set to 0.7 D and AGC was set to 250% with a max injection time of 86 ms.

### Database Search

The raw files were searched using FragPipe (version 19.1). The FragPipe pipeline relies on MSFragger (version 3.7)^50,51^ for peptide identification and Philosopher (version 4.8.0)^52^ for FDR filtering and downstream processing. The chimeric Uniprot protein database used here contains canonical isoforms for both Mouse (Mus musculus; accessed 02/2023) and Human (Homo sapiens, accessed 02/2019). The workflow used in FragPipe followed default TMT-16 plex parameters, used for both TMT-16 and TMT-18 experimental design. Briefly, precursor mass tolerance was −20 to 20 ppm, fragment mass tolerance of 20 ppm, mass calibration and parameter optimization were selected, and isotope error was set to −1/0/1/2/3. Enzyme specificity was set to strict-trypsin and up to two missing trypsin cleavages were allowed. Peptide length was allowed to range from 7 to 50 and peptide mass from either 200 to 5,000 Da. Variable modifications that were allowed in our search included: oxidation on methionine, N-terminal acetylation on protein, and N-terminal acetylation on peptide, with a maximum of 3 variable modifications per peptide. Peptide Spectral Matches were validated using Percolator.^53^ The false discovery rate (FDR) threshold was set to 1% and protein and peptide abundances were quantified using Philosopher for downstream analysis.

### Protein Quantitation and Quality Control

The protein abundances are first normalized by scaling total protein signal within each channel for each specific sample to the maximum channel-specific total signal. We then used a tunable median polish approach, TAMPOR, to remove technical batch variance in the proteomic data, as previously described^54^ TAMPOR is utilized to remove intra-batch and inter-batch variance while preserving meaningful biological variance in protein abundance values, normalizing to the median of selected intra-batch samples. This approach is robust to outliers and columns with up to 50% values missing. If a protein had more than 50% samples with missing values, it was removed from the matrix. No imputation of missing values was performed for any cohort. For the current data, TAMPOR leverages the median protein abundance from the pooled Global Internal Standard (GIS) TMT channels as the denominators in both factors to normalize sample-specific protein abundances across the 2 batches of samples.

### Proteomic data processing and analysis

Normalized abundances were log_2_-transformed prior to modeling. Differential abundance modeling: Differential protein abundance was assessed in R (v4.4.2) using limma (3.62.2) with empirical Bayes moderation. Condition Monocyte versus iMG was modeled as the primary effect; when multiple samples derived from the same donor were present, within-donor correlation was estimated with duplicateCorrelation(). Significance was defined as FDR (Benjamini–Hochberg) < 0.05, with effect-size thresholds of |log_2_FC| > 0.5 or > 1 as indicated. Quality control and visualization: Global data structure and sample comparability were evaluated by principal component analysis (PCA) and hierarchical clustering. Heatmaps were generated with pheatmap (1.0.13 using row-wise z-scores of log_2_ abundances and annotated by condition. Volcano plots were produced with EnhancedVolcano (1.24.0) to display log fold change versus significance. *Violin plots:* For each protein, log2- transformed abundances were visualized as violin plots using ggplot2. Violin bodies show kernel density estimates; centered boxplots display the median and interquartile range. Proteins were ordered by the difference in mean expression between conditions (Monocyte − iMG). For each gene, differences between iMG and Monocyte were assessed with a two-sided, unpaired Wilcoxon rank-sum test (*wilcox.test via ggpubr::compare_means*). The resulting p-values are shown in Figure 3c.

### MoSeq

3D depth videos were recorded in a circular 17” diameter open-field arena (OFA) using a Microsoft Axure Kinect camera. Before recording, the mice were habituated in the examination room for 45 min. During the behavioral recording, the mice were freely moving in the arena for 30 minutes, then put back to its home cape. Between recording sessions, the area was cleaned according to the Moseq protocol. The recorded videos were analyzed using the MoSeq framework, available at http://moseq4all.org. MoSeq is an unsupervised machine learning framework that process and analyze animal spontaneous behavior into sub-second, repeated and modulated segments called “syllables”. The pipeline first processed the video such that the mouse was in the center of the video and the mouse was pointing right. The processed videos then went through dimensionality reduction and modeling to get the frame-by-frame behavioral labels. Syllable usages were aggregated by experimental group in the analysis. Syllable usage differences were identified using Kruskal-Wallis test and a post hoc Dunn’s two-sided test with permutation, using Bejamini-Hochberg FDR (α=0.05).^55^ Linear discriminant analysis (LDA) is a method that finds the linear combination of inputs to maximize group separation. To visualize the behavioral difference between the xenografted mice, LDA took syllable usages and experimental group-day pair as input to learn the low dimensional embedding. The position heatmap was computed by aggregating the mouse centroid positions and the heatmap represents the percentages of time. Distance traveled was computed by summing the between frame Euclidean distance between the mouse centroids in two consecutive frames within a given duration.

### Marble Burying

Standard polycarbonate mouse cages were fitted with filter-top covers and filled with 5mm of fresh, unscented bedding material. 15 glass marbles (3 rows, equally spaced, with 5 marbles per row) were placed at the surface of the bedding. Mice in their home cages were then transferred to the behavioral room to acclimate. After 45 min, each mouse was transferred to the behavioral cage. After 30 min, mice were transferred back to their individual home cages, and the number of buried marbles (>50% below the surface of the bedding) were scored by three blinded observers as previously described^56^ whose scores were averaged per mouse.

### Nesting

Mice were transferred to a standard polycarbonate mouse cage with filter-top cover containing a single nestlet. After 24 hours, nest building was scored on a scale of 1–5 as previously described^57^ by three blinded observers whose scores were averaged per mouse per day. Mice were then transferred to a new cage with a new nestlet and similarly scored a second time after 24 hours. Mice were then transferred to a new cage for the third time with a new nestlet and similarly scored after 24 hours. Final scores were calculated by averaging nesting score across three days per mouse.

### Statistic Calculations

GraphPad Prism 9 was used to perform statistical tests and generate *P* values. We used standard designation of *P* values throughout the Figures (ns = not significant or *p*≥0.05; *p<0.05; **p<0.01; ***p<0.001; ****p<0.0001). Values are represented as average mean ± SEM. Details of number of replicates and the specific statistical test used are provided in the individual figure legends. Data represented for all antibodies calculated from an average of 2–3 matched coronal sections per animal. For quantification of NeuN density a Grubbs outlier test identified one statistical outlier which was removed. Because group distributions of NeuN counts deviated from normality, we utilized a non-parametric Kruskal–Wallis test followed by Dunn’s multiple comparisons.

## Supplementary Material

Supplementary Files

This is a list of supplementary files associated with this preprint. Click to download.

• DavtyanTable1DEGs.xlsx

• DavtyanTable2DEPs.xlsx

• DavtyanTable3KEGGenrichment.xlsx

• ExtendedDataFigures.docx

## Data Availability

The spatial RNA-seq datasets are available through the GEO Super Series accession number (will be added prior to publication). Proteomic data will be made available via Further information and requests for resources and reagents should be directed to and will be fulfilled by Mathew Blurton-Jones (mblurton@uci.edu).
